# Totally laparoscopic resolution of gallstone ileus: A case report

**DOI:** 10.1016/j.ijscr.2021.106682

**Published:** 2021-12-11

**Authors:** Nicolás H. Dreifuss, Francisco Schlottmann, Antonio Cubisino, Alberto Mangano, Carolina Baz, Mario A. Masrur

**Keywords:** Gallstone ileus, Intestinal obstruction, Laparoscopic surgery, Enterolithotomy, Case report

## Abstract

**Introduction:**

Gallstone ileus is an uncommon complication of long-term cholelithiasis. Emergent operations for gallstone ileus are associated with high postoperative morbidity. When feasible, the minimally invasive approach might help to improve the postoperative outcomes.

**Presentation of case:**

A 63-year-old female was admitted for abdominal pain and vomiting. Computed tomography (CT) scan showed a cholecystoduodenal fistula and a 5 × 3 cm gallstone in the jejunum causing obstruction. An emergent laparoscopy was performed, and a gallstone was found inside the jejunum 40 cm distal to the ligament of Treitz. The 5 cm gallstone was extracted through an antimesenteric enterotomy. The jejunum was then closed transversally using interrupted sutures. The postoperative course was uneventful, and the patient was discharged on postoperative day 3.

**Discussion:**

Surgery is the mainstream treatment for gallstone ileus. Multiple operations and surgical approaches have been described: enterolithotomy (EL), one-stage surgery (EL, cholecystectomy, and fistula closure), bowel resection, and two-stage surgery (EL and delayed cholecystectomy with fistula closure). The choice of the procedure depends on the patient's characteristics, comorbidities, and experience of the surgical team.

**Conclusion:**

In the emergency setting, a simple enterolithotomy with primary closure seems to be the optimal approach to solve the intestinal obstruction with low postoperative morbidity. The laparoscopic approach to gallstone ileus results in additional benefits for patients' recovery.

## Highlights

• Gallstone ileus is an uncommon complication of long-term cholelithiasis.
• CT scan is the best imaging modality for diagnosis and preoperative planning.
• Laparoscopic enterolithotomy is safe and results in low postoperative morbidity.

## 1 Introduction

Gallstone ileus results from the impaction of one or more stones along the gastrointestinal tract. It occurs in 0.3–1.5% of patients with cholelithiasis and accounts for 1–4% of all mechanical small bowel obstructions in the general population and 25% in those over 65 years old [1], [2], [3], [4]. Gallstone ileus is preceded by chronic gallbladder inflammation and the formation of adhesions to the duodenum, more frequently. This eventually leads to a biliary-enteric fistula development and passage of stones to the small bowel. Small stones (<2.5 cm) are eliminated through the rectum. In contrast, larger stones (>4–5 cm) become impacted in narrow areas of the gastrointestinal tract causing a mechanical obstruction. Surgery is the cornerstone of gallstone ileus treatment. Historically, these cases were managed by an open approach resulting in significant postoperative morbidity and mortality (7–30%) [5]. In recent years, the minimally invasive approach has become more common resulting in better postoperative outcomes [6]. We describe a gallstone ileus case that was solved with a minimally invasive approach. This case report has been reported in line with the SCARE 2020 criteria [7].

## 2 Presentation of case

**Fig. 1:**
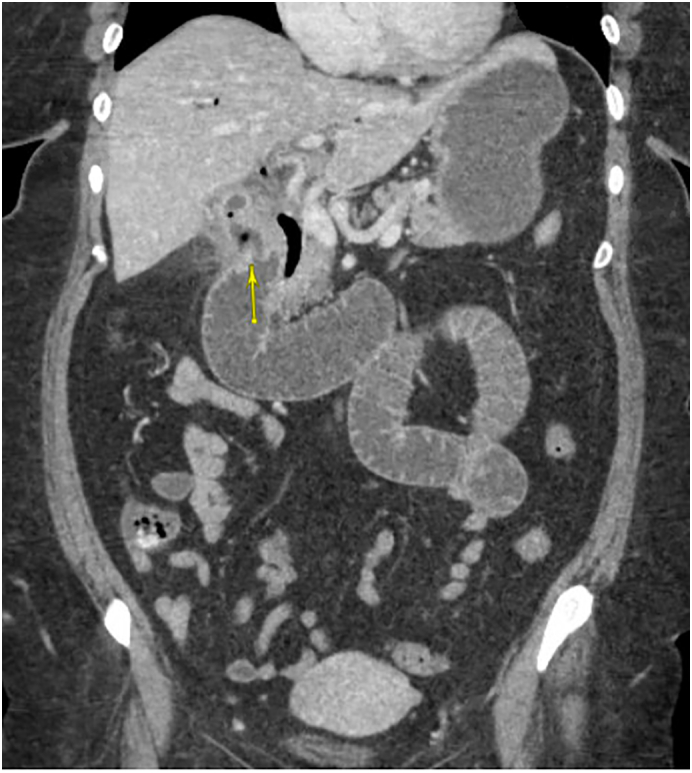
Computed tomography of the abdomen showing pneumobilia and a fistulous track between the gallbladder and duodenum.

**Fig. 2:**
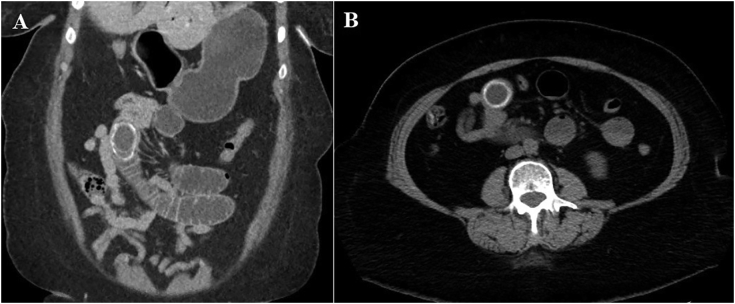
Computed tomography of the abdomen showing (A: coronal section, B: axial section) a 5 cm stone with peripheral calcifications obstructing the jejunum.

A 63-year-old female patient with a past medical history of hypertension, hyperlipidemia, and gastroesophageal reflux disease was admitted to the emergency department for epigastric and right upper quadrant abdominal pain, nausea, and several episodes of emesis. She had no previous abdominal operations. Physical examination revealed tenderness to palpation in the epigastric region but no signs of peritoneal irritation. Laboratory tests showed leukocytosis (12,200/mm^3^) with neutrophilia (74%). Multidetector multiplanar CT scan of the abdomen and pelvis after the administration of 100 cm^3^ of intravenous contrast showed an inflamed gallbladder fistulized to the adjacent duodenum, pneumobilia (Fig. 1), and a 5 × 3 cm gallstone in the mid jejunum causing bowel obstruction (gallstone ileus) (Fig. 2A–B).

**Fig. 3:**
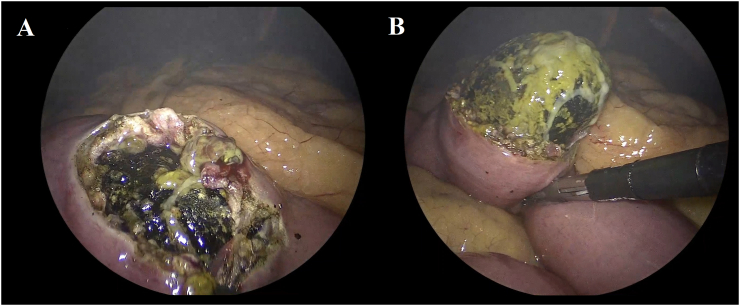
Intraoperative picture showing the enterotomy in the jejunum antimesenteric border (A) and stone extraction (B).

**Fig. 4:**
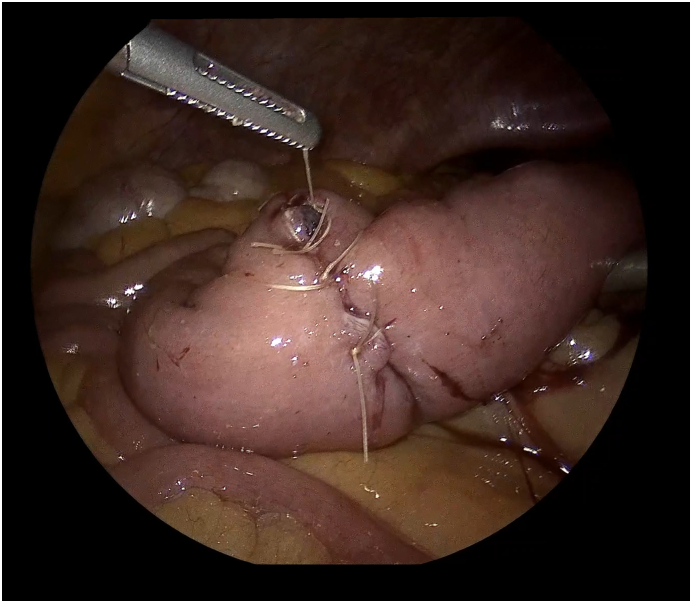
Intraoperative picture showing the intracorporeal transversal enterotomy closure.

The patient underwent an emergent laparoscopy using a 3-trocar technique. The Treitz ligament was identified and 40 cm distally a gallstone was evidenced inside a dilated jejunum loop. Normal caliber jejunal loops distal to the gallstone were identified. An antimesenteric enterotomy proximal to the gallstone was performed using hook electrocautery and a 5 cm gallstone was extracted (Fig. 3A–B). The gallstone was exteriorized from the abdomen through the 12 mm trocar with an EndoCatch device. The jejunum was then closed transversally using interrupted sutures of 3-0 Vycril (Fig. 4). The postoperative course was uneventful, and the patient was discharged home on postoperative day 3. After 4 months of follow-up the patient is doing well and remains asymptomatic.

## 3 Discussion

Gallstone ileus is an uncommon long-term complication of cholelithiasis. It results from the passage of one or more stones through a biliary-enteric fistula which ultimately causes a small bowel obstruction. Generally, the obstruction is at the level of the terminal ileum due to its narrow lumen. Less frequently, the stone cause blockage of the proximal jejunum (like the present case) or even the duodenum (Bouveret syndrome). Clinical presentation is similar to other causes of mechanical bowel obstruction. CT scan is the best imaging modality to accurately diagnose gallstone ileus. Besides the classic Rigler's triad identification (ectopic calcified stone, pneumobilia, and intestinal obstruction), a CT scan can show partially calcified stones, assess the specific site of the obstruction (critical for the operative strategy), and characterize the biliary-enteric fistula [8].

Historically, treatment of gallstone ileus was associated with elevated morbidity and mortality (7–30%), mainly due to the affected population (elderly patients) and delayed diagnosis [5]. Surgery is considered the gold standard treatment for gallstone ileus. However, different techniques have been described: enterolithotomy (EL), one-stage surgery (EL, cholecystectomy, and fistula closure), bowel resection, and two-stage surgery (EL and delayed cholecystectomy with fistula closure). Although treating the fistula and the small bowel obstruction simultaneously reduces the risk of recurrence and gallbladder malignancy, it is technically more demanding and has been associated with increased mortality when compared with simple EL [4], [5]. This procedure might only be considered in young and fit patients. Similarly, the two-stage procedure is indicated in fit patients with persistent biliary symptoms (abdominal pain, cholecystitis, and malabsorption).

Simple enterotomy and stone extraction is the treatment of choice for most patients in the emergency setting. Halabi et al. reported a significant lower mortality in patients who underwent simple EL (5%) when compared to other alternatives such as one-stage surgery (7%), bowel resection alone (13%), and bowel resection with fistula closure (7%) [5]. Simple EL should be combined with a meticulous exploration of the entire small bowel to rule out additional stones (reported in about 25% of patients) which might cause recurrent gallstone ileus [5]. Potential recurrence of an intestinal obstruction is the main drawback of not performing a cholecystectomy with biliary-enteric fistula repair. Recurrence occurs in approximately 8.3% of the cases and usually presents during the first 6 months following the initial operation [9]. Once the emergency is solved, both observation and performing a delayed cholecystectomy and closure of the biliary-enteric fistula are valid options. Spontaneous closure of the fistula can occur in patients with stone-free gallbladder and a patent cystic duct. On the other hand, fistula persistence can lead to cholangitis and recurrent gallstone ileus. The possibility of gallstone ileus and biliary symptoms recurrence must be weighed against the risks of the reoperation. A recent nationwide analysis found a 41.2% morbidity and 2.9% mortality rate in patients who underwent elective fistula closure and cholecystectomy [5].

Laparoscopic approach to gallstone ileus, when feasible, reduces postoperative morbidity and provides a faster recovery [5], [6], [10]. Despite the benefits, a review of 3268 gallstone ileus cases from the Nationwide Inpatient Sample showed that only 10% of the patients were managed laparoscopically [5]. Due to the presence of dilated small bowel, creation of pneumoperitoneum, trocars' insertion, and bowel manipulation carry higher risks. Depending on the experience of the surgical team, the EL and enterotomy closure could be performed either intracorporeal or extracorporeal. Intracorporeal anastomosis is desirable because it prevents extending one of the laparoscopic trocar incisions resulting in reduced wound morbidity.

This study is limited by its nature (case report) and lack of long-term follow-up. Larger series on the laparoscopic resolution of gallstone ileus and comparisons between approaches are required to draw more definitive conclusions.

## 4 Conclusion

Surgical management of gallstone ileus is challenging. The choice of the procedure depends on the patient's characteristics, comorbidities, and experience of the surgical team. Laparoscopic EL results in low postoperative mortality and faster recovery when performed in selected patients by surgeons with vast experience in minimally invasive surgery.

## Consent

Written informed consent was obtained from the patient for publication of this case report and accompanying images. A copy of the written consent is available for review by the Editor-in-Chief of this journal on request.

## Provenance and peer review

Not commissioned, externally peer-reviewed.

## Ethical approval

This study was performed in accordance with the Declaration of Helsinki, the institutional review board (IRB) approval of our institution (IRB#2021-0520).

## Funding

This research did not receive any specific grant from funding agencies in the public, commercial, or not-for-profit sectors.

## Guarantor

NHD.

## Research registration number

N/A.

## CRediT authorship contribution statement

**Conception and design:** NHD, FS, AC, MM. **Data acquisition, analysis and interpretation of data:** NHD, CB, AC, AM. **Drafting of manuscript, and critical revision of manuscript:** NHD, FS, AC, AM, CB, MM. Operation performed by: FS. All authors read and approved the final manuscript.

## Declaration of competing interest

The authors have no conflicts of interest or financial ties to disclose.

## References

[1] Kurtz R.J., Heimann T.M., Kurtz A.B. (1983). Gallstone ileus: a diagnostic problem. Am. J. Surg..

[2] Kasahara Y., Umemura H., Shiraha S., Kuyama T., Sakata K., Kubota H. (1980). Gallstone ileus. Review of 112 patients in the Japanese literature. Am. J. Surg..

[3] Brockis J.G., Gilbert M.C. (1957). Intestinal obstruction by gall-stones; a review of 179 cases. Br. J. Surg..

[4] Reisner R.M., Cohen J.R. (1994). Gallstone ileus: a review of 1001 reported cases. Am. Surg..

[5] Halabi W.J., Kang C.Y., Ketana N. (2014). Surgery for gallstone ileus: a nationwide comparison of trends and outcomes. Ann. Surg..

[6] Moberg A.C., Montgomery A. (2007). Laparoscopically assisted or open enterolithotomy for gallstone ileus. Br. J. Surg..

[7] Agha R.A., Franchi T., Sohrabi C., Mathew G., for the SCARE Group (2020). The SCARE 2020 guideline: updating consensus Surgical CAse REport (SCARE) Guidelines. International Journal of Surgery.

[8] Chuah P.S., Curtis J., Misra N. (2017). Pictorial review: the pearls and pitfalls of the radiological manifestations of gallstone ileus. Abdom. Radiol..

[9] Doogue M.P., Choong C.K., Frizelle F.A. (1998). Recurrent gallstone ileus: underestimated. Aust. N. Z. J. Surg..

[10] Shiwani M.H., Ullah Q. (2010). Laparoscopic enterolithotomy is a valid option to treat gallstone ileus. JSLS.

